# Supplementary material for: Influence of Gut Microbiota on Metabolism of Bisphenol A, a Major Component of Polycarbonate Plastics
Source: Toxics. 2023 Mar 31;11(4):340. doi: 10.3390/toxics11040340 (PMC10144690; doi:10.3390/toxics11040340)
Supplement: Supplementary file 1 [file toxics-11-00340-s001.zip › toxics-2296237-supplementary.pdf]

SUPPORING INFORMATION

Influence of Gut Microbiota on Metabolism of Bisphenol A, A Major Component of Polycarbonate Plastics

Weili Mao [1], Lingling Mao [2], Feifei Zhou [3], Jiafeng Shen [3], Nan Zhao [2], Hangbiao Jin [2,*], Jun Hu [2] and Zefu Hu [1,*]

[1] Department of Pharmacy, Quzhou Affiliated Hospital of Wenzhou Medical University, Quzhou People's Hospital, Quzhou, Zhejiang 310032, China.

[2] Key Laboratory of Microbial Technology for Industrial Pollution Control of Zhejiang Province, College of Environment, Zhejiang University of Technology, Hangzhou, Zhejiang 310032, China.

[3] CAS Testing Technical Services Jiaxing Co., Jiaxing 314000, China.

Extraction Method. Rat blood samples, spiked with internal standards (5 ng each), were extracted by adding 5 mL of methanol to whole blood samples (500 μL). Following the addition of methanol, the mixtures were shaken for 20 min at 4 °C, sonicated for 30 min in ice-bath, and centrifuged for 10 min at 3000 g to separate the supernatant. The residual was extracted twice more with the same method. All of the obtained supernatants were combined (~15 mL, in total) and reduced to ~3 mL under gentle nitrogen evaporation, followed by being passed through the ENVI-Carb SPE cartridges (Supelclean® 250 mg/3 mL; Sigma-Aldrich, ON, Canada). These SPE cartridges were sequentially preconditioned with methanol (6 mL) and water (6 mL). After purification, the eluents were evaporated to dryness under nitrogen, and then reconstituted with 100 μL of 50% methanol/water.

Urine samples were extracted following the method of Liao et al.[1] In detail, rat urine samples (50 μL), spiked with internal standards (5 ng each), were mixed with pure water (950 μL, containing 0.1% formic acid) for dilution, and then extracted using solid-phase extraction. Diluted urine samples were loaded onto the Sep-Pak C18 cartridges (500 mg/6 cc; Waters, Milford, USA), which was conditioned with 6 mL of methanol and 6 mL of 0.1% formic acid/water before use. After the loading of urine, the cartridges were washed with 4 mL of 15% methanol/water. Finally, 6 mL of methanol (containing 1% NH_4OH) was used to elute the target analytes retained in the cartridges. Such eluents were

evaporated to dryness under nitrogen, and then reconstituted with 200 μL of 50% methanol/water.

Feces samples were freeze-dried in a vacuum (at around –50 °C for 36 h), ground, and then extracted using a solvent composition of 90% methanol/water. In total, 5 mL of 90% methanol/water was added to the dried feces samples (0.05 g), which had been previously spiked with internal standards (5 ng each). After that, the feces samples were homogenized using a vortexing process for 5 min, sonicated for 30 min, and centrifuged for 10 min to separate the supernatant. The residual feces were extracted once more using the same method. The two supernatants were mixed and further purified using ENVI-Carb SPE cartridges, as described above. Finally, the residual of feces extract was reconstituted with 100 μL of 50% methanol/water.

Spike and Recovery Experiments. Extraction recoveries of target analytes from the rats' blood, urine, and feces were determined by spiking 500 μL of rat blood, 100 μL of rat urine, or 50 mg of rat feces from unspiked SD rats (n = 5) with 5 ng BPA, 5 ng BPA-G, 5 ng BPA-S, 1 ng BPA-BG, and 1 ng BPA-DS. All these fortified samples were stored at –80 °C for 24 hours, and then extracted using the above-mentioned methods. Results of the extraction recovery experiments are provided in Tables S2.

Table S1. Names, Abbreviations, Parent Ions, and Daughter Ions of Bisphenol A and Its Conjugated Metabolites.

Full name	Abbreviation	MRM transition	
		Quantification (*m*/*z*)	Qualification (*m*/*z*)
	Native standard		
**Bisphenol A mono-*β*-d-glucuronide**	BPA-G	403/227	403/113
**Bisphenol A bis-*β*-d-glucuronide**	BPA-BG	579/403	579/175
**Bisphenol A mono-sulfate**	BPA-S	307/227	307/212
**Bisphenol A di-sulfate**	BPA-DS	387/307	387/227
**Bisphenol A**	BPA	227/133	227/212
	Isotopically-labeled standard		
**$^{13}C_{12}$-Bisphenol A**	$^{13}C_{12}$-BPA	239/224	
**$^{13}C_{12}$-Bisphenol A mono-*β*-d-glucuronide**	$^{13}C_{12}$-BPA-G	415/175	
**D_6-BPA mono-sulfate**	D_6-BPA-S	313/215	

Table S2. Extraction Recoveries (%; n = 5) of BPA and Its Conjugated Metabolites in Blood, Feces, and Urine of SD Rats. Rat Blood, Feces, and Urine Samples were Spiked at 50 ng/mL, 50 ng/g, and 50 ng/mL, respectively.

	**Blood**		**Feces**		**Urine**	
	**Mean**	**SD**	**Mean**	**SD**	**Mean**	**SD**
**BPA**	92	6	104	10	81	12
**BPA-S**	72	9	114	17	87	10
**BPA-G**	93	5	105	10	79	12
**BPA-DS**	73	3	94	9	92	5
**BPA-BG**	81	11	90	14	74	6

Table S3. LODs of BPA and Its Conjugated Metabolites in Blood, Feces, and Urine of SD Rats.

	Blood (ng/mL)	Feces (ng/g)	Urine (ng/mL)
**BPA**	0.047	0.052	0.039
**BPA-S**	0.088	0.071	0.15
**BPA-G**	0.052	0.077	0.073
**BPA-DS**	0.061	0.039	0.066
**BPA-BG**	0.050	0.059	0.092

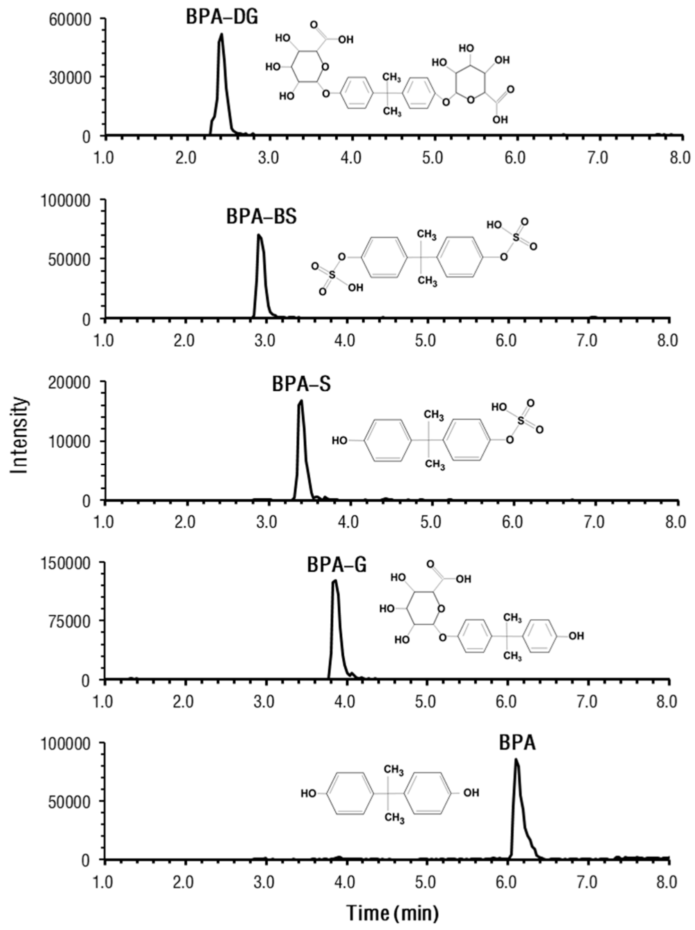

Figure S1. Typical chromatograms and molecular structures of BPA and its conjugate metabolites.

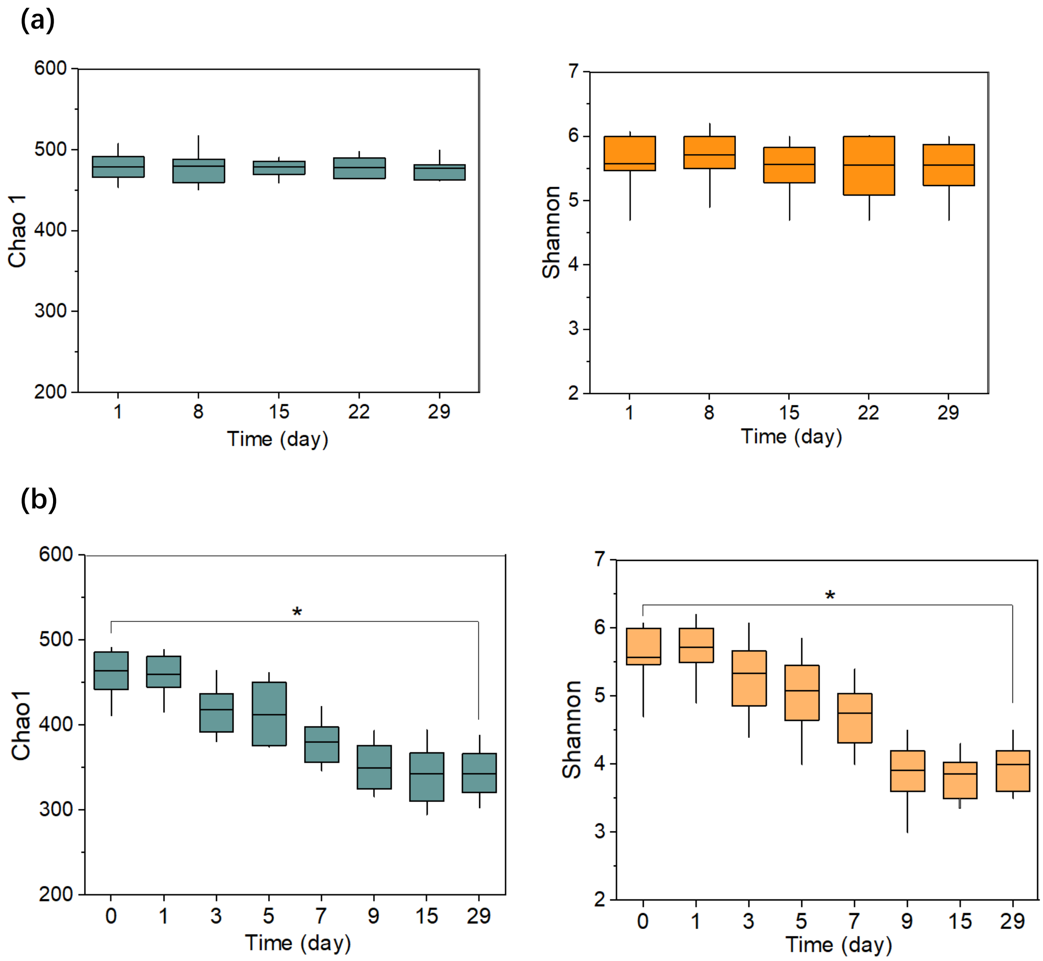

Figure S2. Change in the alpha diversity (Chao1 estimator and Shannon index) of rat gut microbiota with time, following (a) interval and (b) continuous oral BPA exposure. An independent sample *t*-test was used to determine the differences between the Chao1 estimator and Shannon index from day 0 to day 28. The ⁎ means that the difference was significant ($p < 0.01$).

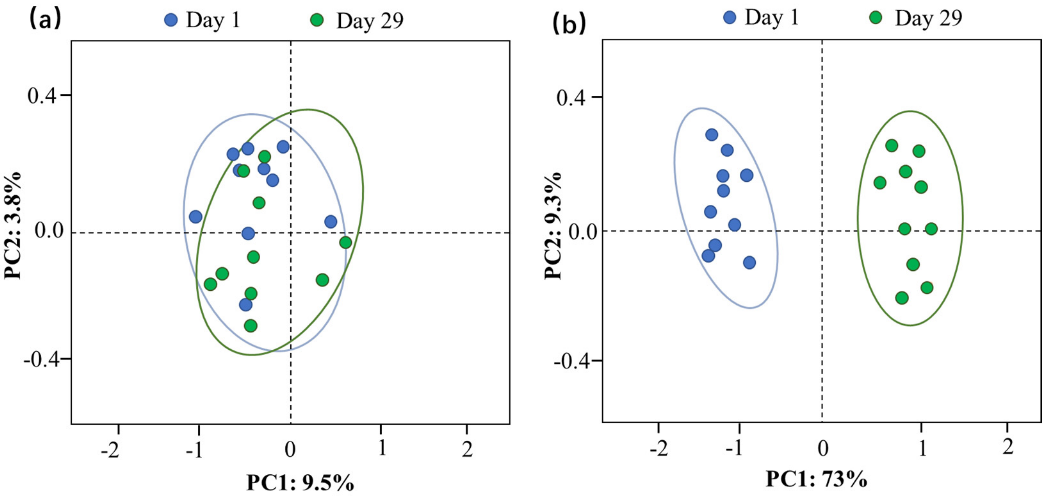

Figure S3. Principal coordinate analysis of the gut microbiome patterns of rats at day 1 and 29 following (a) intervallic and (b) continuous oral BPA exposure.

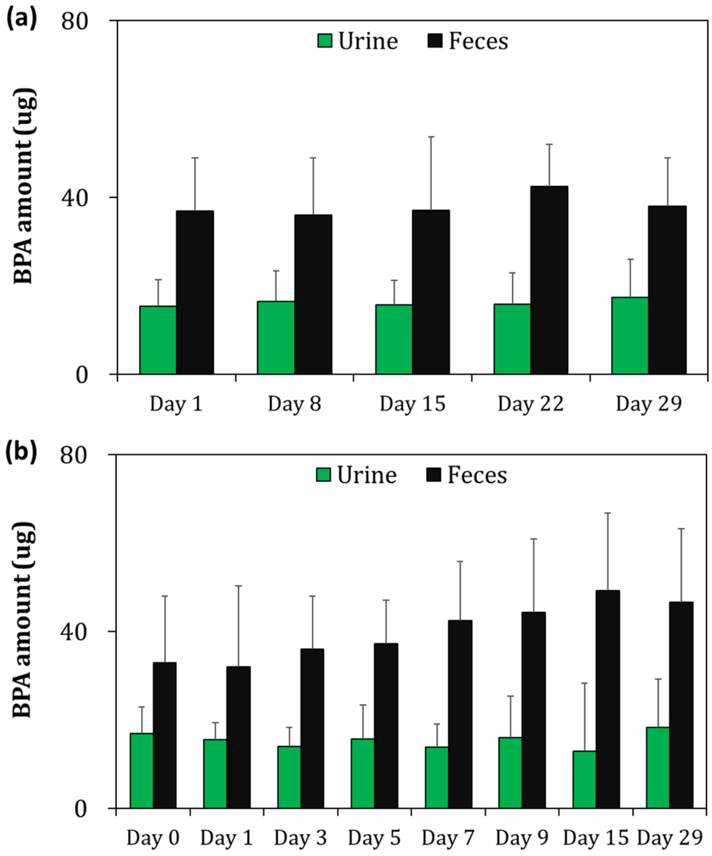

Figure S4. Amount of BPA (μg; mean ± SD) excreted via the rats' urine and feces, following (a) intermittent and (b) continuous BPA exposure. Note that BPA conjugates were transferred to BPA based on the method of previous studies.[2,3] BPA excreted in glucuronide form = BPA-G × 0.5614; BPA excreted in sulfate form = BPA-S × 0.7404. The factors 0.5614 and 0.7404 are the ratios of the molecular weight of BPA to the molecular weights of BPA-G and BPA-S, respectively.

Reference

1. Liao, C.; Kannan, K., Determination of free and conjugated forms of bisphenol A in human urine and serum by liquid chromatography-tandem mass spectrometry. *Environ Sci Technol* **2012,** *46*, (9), 5003-9.

2. Gerona, R. R.; Pan, J.; Zota, A. R.; Schwartz, J. M.; Friesen, M.; Taylor, J. A.; Hunt, P. A.; Woodruff, T. J., Direct measurement of Bisphenol A (BPA), BPA glucuronide and BPA sulfate in a diverse and low-income population of pregnant women reveals high exposure, with potential implications for previous exposure estimates: a cross-sectional study. *Environmental*

Health **2016,** *15*.
3. Gerona, R. R.; Woodruff, T. J.; Dickenson, C. A.; Pan, J.; Schwartz, J. M.; Sen, S.; Friesen, M. W.; Fujimoto, V. Y.; Hunt, P. A., Bisphenol-A (BPA), BPA Glucuronide, and BPA Sulfate in Midgestation Umbilical Cord Serum in a Northern and Central California Population. *Environ Sci Technol* **2013,** *47*, (21), 12477-12485.
